# Pharmaceutical expenditure forecast model to support health policy decision making

**DOI:** 10.3402/jmahp.v2.23740

**Published:** 2014-06-04

**Authors:** Cécile Rémuzat, Duccio Urbinati, Åsa Kornfeld, Anne-Lise Vataire, Laurent Cetinsoy, Samuel Aballéa, Olfa Mzoughi, Mondher Toumi

**Affiliations:** 1 Creativ-Ceutical France, 215 rue du Faubourg Saint-Honoré, 75008 Paris, France; 2 University Claude Bernard Lyon I, UFR d'Odontologie, 11 rue Guillaume Paradin, 69372, Lyon, Cedex 08, France

**Keywords:** forecast model, pharmaceutical expenditure, health policy, generic, biosimilar, innovative medicine

## Abstract

**Background and objective:**

With constant incentives for healthcare payers to contain their pharmaceutical budgets, modelling policy decision impact became critical. The objective of this project was to test the impact of various policy decisions on pharmaceutical budget (developed for the European Commission for the project ‘European Union (EU) Pharmaceutical expenditure forecast’ – http://ec.europa.eu/health/healthcare/key_documents/index_en.htm).

**Methods:**

A model was built to assess policy scenarios’ impact on the pharmaceutical budgets of seven member states of the EU, namely France, Germany, Greece, Hungary, Poland, Portugal, and the United Kingdom. The following scenarios were tested: expanding the UK policies to EU, changing time to market access, modifying generic price and penetration, shifting the distribution chain of biosimilars (retail/hospital).

**Results:**

Applying the UK policy resulted in dramatic savings for Germany (10 times the base case forecast) and substantial additional savings for France and Portugal (2 and 4 times the base case forecast, respectively). Delaying time to market was found be to a very powerful tool to reduce pharmaceutical expenditure. Applying the EU transparency directive (6-month process for pricing and reimbursement) increased pharmaceutical expenditure for all countries (from 1.1 to 4 times the base case forecast), except in Germany (additional savings). Decreasing the price of generics and boosting the penetration rate, as well as shifting distribution of biosimilars through hospital chain were also key methods to reduce pharmaceutical expenditure. Change in the level of reimbursement rate to 100% in all countries led to an important increase in the pharmaceutical budget.

**Conclusions:**

Forecasting pharmaceutical expenditure is a critical exercise to inform policy decision makers. The most important leverages identified by the model on pharmaceutical budget were driven by generic and biosimilar prices, penetration rate, and distribution. Reducing, even slightly, the prices of generics had a major impact on savings. However, very aggressive pricing of generic and biosimilar products might make this market unattractive and can be counterproductive. Worth noting, delaying time to access innovative products was also identified as an effective leverage to increase savings but might not be a desirable policy for breakthrough products. Increasing patient financial contributions, either directly or indirectly via their private insurances, is a more likely scenario rather than expanding the national pharmaceutical expenditure coverage.

Policy makers of the European Union (EU) member states devise pharmaceutical policies that aim at achieving efficient spending for healthcare payers. They set policies directed both at rewarding innovation and favoring generic and biosimilar markets. Innovation is one of the pillars of the pharmaceutical sector via its acknowledgement through intellectual property rights. It is of key importance that the research based pharmaceutical industry gets the right rewards to invest in high risk development products. The generic and biosimilar markets play an important role in keeping healthcare public budget under control while sparing for innovative medicines (1, 2).

In the context of the current global economic downturn and the constant motivation for healthcare payers to contain the pharmaceutical budgets, modelling policy decision impact has become critical for the EU member states and for the European regulatory bodies.

The objective of this project was to study the impact of various policy changes on the seven EU member states’ pharmaceutical budget. This project was performed for the European Commission (‘EU Pharmaceutical expenditure forecast’ – http://ec.europa.eu/health/healthcare/key_documents/index_en.htm)

## Method

A pharmaceutical expenditure forecast model was developed with the aim of supporting policy decision makers assessing the effect of changes in national pharmaceutical policies on pharmaceutical budget. The study was supervised and validated by a panel of six experts, with strong experience in market access of healthcare products and healthcare policies.

**Fig. 1:**
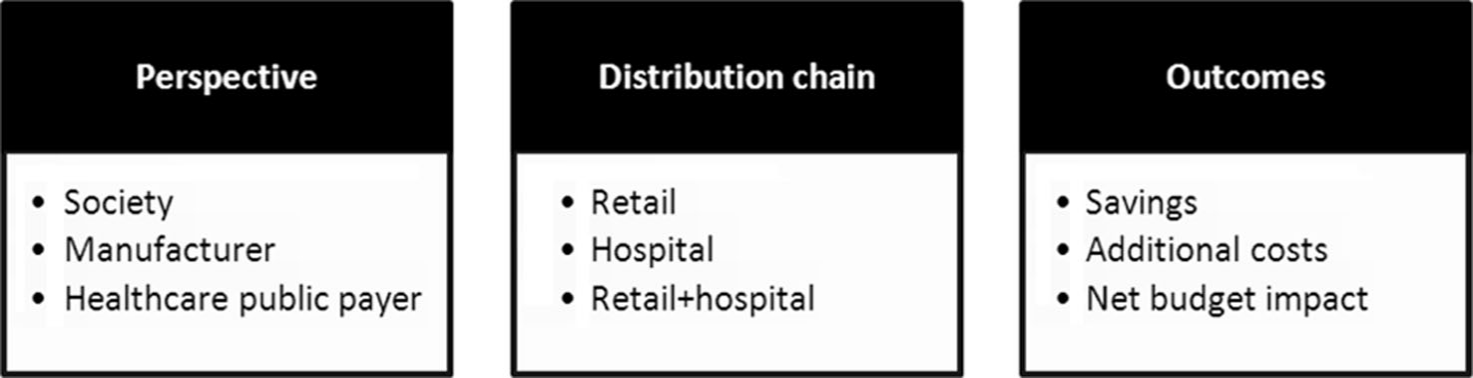
Description of model characteristics.

The forecast model included a model for generics and biosimilars and a model for the newly approved innovative pharmaceutical products. The model computed the pharmaceutical budget impact of genericization and of new innovative drug entries over the 2012–2016 time period (using drugs’ sales values for 2011 extracted from the IMS database) for the seven EU member states, namely France, Germany, Greece, Hungary, Poland, Portugal, and the United Kingdom. The model was developed based on three perspectives (healthcare public payer, society, and manufacturer), several types of distribution chains (retail, hospital, retail and hospital combined), and several outcomes (savings due to products going off-patent, additional costs due to new innovative products, and net budget impact)^1^ (Fig. 1). Only results from the healthcare public payer perspective are presented in this manuscript. Probabilistic and deterministic sensitivity analyses were carried out regarding the intrinsic uncertainty surrounding the estimations. The detailed description of the model, including probabilistic and deterministic sensitivity analyses, and the forecasting exercise are presented in two separate articles, ‘Novel methodology for pharmaceutical expenditure forecast’ and ‘EU pharmaceutical expenditure forecast’.

The model parameters used to assess the pharmaceutical ‘reference’ forecast (later referred to as base case) were obtained from publicly available sources^2^; local experts for Greece, Hungary, Poland, and Portugal; and from the insights of the board of experts. Regarding time to market new drugs, it was assumed for all countries, except for Germany, that due to pricing and reimbursement policies, the sales of new drugs would impact the market 1 year after the date of approval for marketing by the European Medicines Agency. For Germany, the impact was assumed to be from market approval, as companies initiate sales based on a free pricing method for the first year following market approval of the new, innovative drug (Table 1).

**Table 1:** Model parameters used to assess the pharmaceutical ‘reference’ forecast (base case)

	France	Germany	Greece	Hungary	Poland	Portugal	United Kingdom
Brands							
Time to market after marketing authorization (months)	12	0	12	12	12	12	12
Generics – retail chain							
Time to market after marketing authorization (days)	60	0	270	45	180	150	0
Price reduction of the generic versus the original branded product (%)	60	55	60	55	45	60	75
Generic penetration (generics/off-patent brands)-volume uptake (%)	80	85	25	100	85	25	80
Time to reach maximum of generic penetration (months)	36	12	36	18	24	30	12
Impact of generic entry on brand price (%)	20	0	50	0	25	0	0
Biosimilars – Retail chain							
Time to market after marketing authorization (days)	470	180	450	580	540	530	180
Price reduction of the biosimilar versus the original branded product (%)	30	25	25	50	45	30	25
Biosimilar penetration (biosimilar/off-patent brands)-volume uptake (%)	15	25	5	100	25	15	15
Time to reach maximum of biosimilar penetration (months)	36	12	36	18	24	30	12
Impact of biosimilar entry on brand price (%)	10	0	25	0	12	0	0
Generics/Biosimilars-Hospital chain							
Time to market after marketing authorization (days)	0	0	0	0	0	0	0
Price reduction of the generic/biosimilar versus the original branded product (%)	80	80	80	80	80	80	80
Generic/biosimilar penetration (generic-biosimilar/off-patent brands)-volume uptake (%)	100	100	100	100	100	100	100
Time to reach maximum of generic/biosimilar penetration (months)	0	0	0	0	0	0	0
Impact of generic/biosimilar entry on brand price (%)	0	0	0	0	0	0	0
Reimbursement							
Reimbursement rates	69%	90%	80%	67%	62.5%	81.6%	100%

To assess the impact of changes in pharmaceutical policies, nine scenarios were developed in agreement with the board of experts:

1. *Scenario 1*: Pharmaceutical policies of the United Kingdom applied to all countries (refer to Table 1).
2. *Scenario 2*: Change in time to market the new drug after marketing approval to 1 year, for Germany.
3. *Scenario 3*: Change in time to market the new drug after marketing approval to 6 months, for all countries.
4. *Scenario 4*: Change in time to market the new drug after marketing approval to 0 month, for all countries.
5. *Scenario 5*: Change in the level of reimbursement rate to 100%, for all countries.
6. *Scenario 6 and scenario 7*: Change in the price reduction of generics through retail chain to 75 and 85%, respectively, for all countries.
7. *Scenario 8*: Change in the generic penetration rate through retail chain to 100%, for all countries.
8. *Scenario 9*: Change in the distribution chain of biosimilars restricted to hospital, assuming that 100% of biosimilars are distributed through the hospital chain, for all countries. Two sub-scenarios were tested with a price reduction of biosimilar versus the original biologic set at 50 and 80%, respectively.

The net pharmaceutical budget impact assessed in these scenarios was compared to the base case using the model parameters described in Table 1. This base case is presented in Table 2.

**Table 2:** Base case pharmaceutical forecast: Budget impact (per year and on the 2012–2016 period) for total pharmaceutical expenditures from healthcare public payer perspective (in million € (2011€) and expressed as percentage of total 2011 pharmaceutical sales)

Year		Budget impact 2012	Budget impact 2013	Budget impact 2014	Budget impact 2015	Budget impact 2016	Budget impact 2012–2016 (base case)
Country	France	−451 (−1.7%)	−919 (−3.5%)	−1,264 (−4.8%)	−1,511 (−5.7%)	−1,444 (−5.5%)	−5,589
	Germany	−402 (−1.1%)	−561 (−1.6%)	−413 (−1.1%)	−174 (−0.5%)	+719 (+2%)	−831
	Greece	−68 (−1.7%)	−134 (−3.3%)	−164 (−4.1%)	−221 (−5.5%)	−221 (−5.5%)	−808
	Hungary	−27 (−2%)	−31 (−2.3%)	−20 (−1.5%)	−7 (−0.5%)	+1 (+0.1%)	−84
	Poland	−4 (−0.1%)	+9 (+0.3%)	+6 (+0.2%)	+6 (+0.2%)	+24 (+0.7%)	41
	Portugal	−7 (−0.2%)	−21 (−0.6%)	−47 (−1.4%)	−79 (−2.4%)	−89 (−2.7%)	−243
	United Kingdom	−920 (−4.3%)	−1,831 (−8.6%)	−2,210 (−10.4%)	−2,309 (−10.9%)	−2,097 (−9.9%)	−9,367

## Results

### Scenario 1: Pharmaceutical policies of the United Kingdom applied to all countries

While implementing the pharmaceutical policies of the United Kingdom, all countries, except Poland and Hungary, experienced a decrease in their pharmaceutical budget. Germany experienced maximum decrease, with up to 10 times more savings. France and Portugal also enjoyed considerable additional savings under this scenario (2 and 4 times the reference forecast, respectively) (Table 3). The deterministic sensitivity analysis showed that these savings were mainly driven by the increase in generic and biosimilar penetration policies that are in place in the United Kingdom. The new drugs coming into the market had less impact, except for Germany, since delayed entry is an important driver.

**Table 3:** Scenario 1 (Pharmaceutical policies of the United Kingdom applied to all other countries) versus base case–Net budget impact 2012–2016 (2011€) for total pharmaceutical expenditures from healthcare public payer perspective (million €)

Scenario number		Base case	1
Country	France	−5,589	−10,796
	Germany	−831	−8,445
	Greece	−808	−1,199
	Hungary	−84	−76
	Poland	41	42
	Portugal	−243	−951
	United Kingdom	−9,367	−9,367

### Scenario 2: Change in time to market the new drug after marketing approval to 1 year, for Germany

In this second scenario, the time to market new drugs after marketing approval was increased for Germany from zero to 1 year. This scenario exemplified the importance of time to market for new drugs as savings increased from €−831 million to €−5061 million (Table 4).

**Table 4:** Scenario 2 (Change in time to market the new drug after marketing approval to 1 year, for Germany) versus base case–Net budget impact 2012–2016 (2011€) for total pharmaceutical expenditures from healthcare public payer perspective (million €)

Scenario number		Base case	2
Country	France	−5,589	–
	Germany	−831	−5,016
	Greece	−808	–
	Hungary	−84	–
	Poland	41	–
	Portugal	−243	–
	United Kingdom	−9,367	–

### Scenario 3: Change in time to market the new drug after marketing approval to 6 months, for all countries

The EU transparency directive 89/105/EEC (3) aims at a 6-month process for pricing and reimbursement. Therefore, it was interesting to assess the potential impact of enforcing that directive on the studied EU member states. A change in time to market the new drugs after marketing approval to 6 months was applied to all countries in this scenario.

All selected member states, with the exception of Germany, experienced a substantial reduction of their potential savings in the pharmaceutical budget (e.g., from €−5,589 million to €−4,554 million for France, or from €−808 million to €−705 million for Greece). This demonstrates that the enforcement of the European Directive could have a negative impact on public pharmaceutical budget (Table 5).

**Table 5:** Scenario 3 (Change in time to market the new drug after marketing approval to 6 months, for all countries) versus base case–Net budget impact 2012–2016 (2011€) for total pharmaceutical expenditures from healthcare public payer perspective (million €)

Scenario number		Base case	3
Country	France	−5,589	−4,554
	Germany	−831	−3,099
	Greece	−808	−705
	Hungary	−84	−25
	Poland	41	163
	Portugal	−243	−155
	United Kingdom	−9,367	−8,339

### Scenario 4: Change in time to market the new drug after marketing approval to 0 month, for all countries

In this scenario, time to market the new drugs after marketing approval was decreased for all countries, as for Germany, to 0 month. All countries, with the exception of Germany (no change from the baseline scenario), experienced a decrease in their pharmaceutical budget savings (e.g., from €−9,367 million to €−7,083 million for the United Kingdom, or from €−84 million to €+49 million for Hungary). This scenario showed the greatest impact of time to market of new branded products on the net pharmaceutical budget impact (Table 6).

**Table 6:** Scenario 4 (Change in time to market the new drug after marketing approval to 0 month, for all countries) versus base case–Net budget impact 2012–2016 (2011€) for total pharmaceutical expenditures from healthcare public payer perspective (million €)

Scenario number		Base case	4
Country	France	−5,589	−3,363
	Germany	−831	−831
	Greece	−808	−575
	Hungary	−84	49
	Poland	41	337
	Portugal	−243	−49
	United Kingdom	−9,367	−7,083

### Scenario 5: Change in the level of reimbursement rate to 100%, for all countries

A reimbursement rate of 100% was applied in all the countries in this scenario. All countries, with the exception of the United Kingdom (no change from the baseline scenario), experienced an important increase in the net pharmaceutical budget (e.g., €+9,312 versus base case for France and €+686 versus base case for Portugal). In this scenario, healthcare public payers would have to pay the proportion not paid as of now when the reimbursement rate is below 100% (Table 7).

**Table 7:** Scenario 5 (Change in the level of reimbursement rate for all countries to 100%) versus base case–Net pharmaceutical budget impact in 2011 and 2012–2016 for reimbursement rate set at 100% for all countries versus net budget impact 2012–2016 for total pharmaceutical expenditures from healthcare public payer perspective (2011 €, million €)

Scenario number		Base case	5	
			
			Budget impact 2011	Budget impact 2012–2016
Country	France	−5,589	11,823	3,723 (+9,312 versus base case)
	Germany	−831	4,017	3,094 (+3,925 versus base case)
	Greece	−808	1,010	0 (+808 versus base case)
	Hungary	−84	669	544 (+628 versus base case)
	Poland	41	1,954	2,020 (+1,979 versus base case)
	Portugal	−243	741	443 (+686 versus base case)
	United Kingdom	−9,367	0	−9,367 (+0 versus base case)

### Scenario 6 and scenario 7: Change in the price reduction of generics through retail chain to 75 and 85%, respectively, for all countries

In these scenarios, the price reduction of generics through retail chain was set to 75% (as initially set for the United Kingdom, which had the highest price reduction compared to other countries) and 85% (10% higher than the base case value set in the United Kingdom). All countries had an increase in their savings in both scenarios. Savings were greater when the price reduction was set to 85% (e.g., from €−9,367 million to €−10,526 million for the United Kingdom, or from €−84 million to €−201 million for Hungary). These scenarios showed the high sensitivity of savings to generic price (Table 8).

**Table 8:** Scenarios 6 & 7 (Change in the price reduction of generics through retail chain for all countries to 75 and 85%, respectively) versus base case–Net budget impact 2012–2016 (2011€) for total pharmaceutical expenditures from healthcare public payer perspective (million €)

Scenario number		Base case	6	7
Country	France	−5,589	−7,176	−8,234
	Germany	−831	−4,191	−5,871
	Greece	−808	−870	−912
	Hungary	−84	−162	−201
	Poland	41	26	20
	Portugal	−243	−275	−297
	United Kingdom	−9,367	−9,367	−10,526

### Scenario 8: Change in the generic penetration rate through retail chain to 100%, for all countries

Fixing generic penetration rate (through retail chain) to 100%, in scenario 8, increased savings in pharmaceutical budget for France, the United Kingdom, Germany, Portugal, and Greece (e.g., from €−5,589 million to €−6,647 million for France, or from €−243 million to €−628 million for Portugal). Poland experienced only meager additional savings as its initial penetration rate was already 85%, and Hungary did not experience any change (no change from the baseline scenario). This scenario highlights the sensible impact of generic penetration rate on savings (Table 9).

**Table 9:** Scenario 8 (Change in the generic penetration rate through retail chain for all countries to 100%) versus base case–Net budget impact 2012–2016 (2011€) for total pharmaceutical expenditures from healthcare public payer perspective (million €)

Scenario number		Base case	8
Country	France	−5,589	−6,647
	Germany	−831	−2,461
	Greece	−808	−932
	Hungary	−84	−84
	Poland	41	40
	Portugal	−243	−628
	United Kingdom	−9,367	−11,540

### Scenario 9: Change in the distribution chain of biosimilars restricted to hospital, for all countries

The restriction of distribution chain of biosimilars to hospital, in scenario 9, led to increased savings linked to biosimilars in all countries. These savings varied across countries and were higher when the price discount of biosimilar versus the original biologic was set at 80% rather than 50%, but remained substantial in both scenarios. Extra savings were relatively small for the United Kingdom although significant (€353 million when assuming 80% price discount of biosimilar). Important extra savings were observed for France and Germany (around 3 to 4 times the base case savings, that is, €2,190 million for France and €3,504 million for Germany when assuming 80% price discount of biosimilar) (Table 10).

**Table 10:** Scenario 9 (Change in the distribution chain of biosimilars for all countries restricted to hospital) versus base case–total savings linked to biosimilars 2012–2016 (2011 €) from healthcare public payer perspective (million €)

		Assuming 80% price reduction of the biosimilar			Assuming 50% price reduction of the biosimilar		
			
Savings biosimilars 2012–2016		Base case	Scenario 9	Extra savings	Base case	Scenario 9	Extra savings
Country	France	1,127	3,317	2,190	712	2,073	1,361
	Germany	1,014	4,518	3,504	667	2,824	2,157
	Greece	15	225	210	15	141	126
	Hungary	19	205	186	13	128	115
	Poland	125	237	112	78	148	70
	Portugal	222	286	64	139	179	40
	United Kingdom	2,023	2,376	353	1,268	1,485	217

## Discussion

This study highlighted the importance of introducing new regulations on distribution chains to generate savings. This is already implemented in the Netherlands where the Dutch government decided to shift distribution of expensive medicines from retail to hospital right from 2012, starting with TNF-α inhibitors and associated medicine. It was argued that lower prices could be achieved for expensive drugs via hospital negotiations (4, 5). It should be noted that when expensive products are predominantly distributed through hospital pharmacies, such as in the United Kingdom, the expenditure on these products is likely to be lower compared to countries with predominant retail channels such as France and Germany. The high rate of discount (up to 80%), applied in the model for biosimilar products at the hospital level, explains the impact of this distribution. The choice of this discount level was supported by personal experience of the authors based on the discounts applied to the original brand prices of filgrastim and erythropoietin following the launch of their biosimilars (6). In practice though, it is important to consider that at least three biosimilars are necessary on the market to induce companies’ heavy competition through tenders and to observe a fast price erosion. Per se, large savings related to the shift of biosimilars to hospital distribution chain would only happen if competition is significant. On the contrary, significant price cuts on biosimilars might become a disincentive to the production or the commercialization of biosimilars. The threshold that would make the biosimilar market non-attractive and counterproductive remains unclear. More research is warranted to identify the latitude for expanding savings on biosimilars while sustaining a diverse biosimilar industry.

Increasing the penetration of generics and reducing their price is also a powerful leverage to contain the pharmaceutical budget. Combination of both parameters will be of utmost importance, as price reduction in countries with low level of generic penetration would only have a small impact. It could also be justifiable to set the same reimbursement level for generic and brand products (not yet the case for all genericized branded products in all countries). This would be equivalent to assuming a 100% generic penetration rate. However, having brand and generic products at the same price might drive the market toward the brand product that enjoys patient preference. This scenario will be limited in Germany, as generics are already being acquired through tenders. Therefore, it is unlikely that decreasing the price of generics is a realistic option.

This study showed that delaying product reimbursement would be an important lever to reduce the budget impact of new product entries. However, immediate entry of a new drug offers a fundamental benefit for patients who could obtain access to new products from the date of marketing authorization. It is currently reported that time to market is dramatically delayed in several E U countries (7). For instance, in Italy, time to ultimate market access at the regional level is severely delayed. In England, even if there is no delay following marketing authorization, patient access can be considered delayed since actual access is marginal before health technology assessment review is performed by National Institute for Health and Clinical Excellence (NICE) (8, 9). Until NICE issues a recommendation, product uptake remains minimal. In France, the French HTA agency – the French National Authority for Health (*Haute Autorité de Santé-*HAS) and the Economic Committee on Healthcare Products (*Comité Economique des Produits de Santé* – CEPS) – instituted a fast track procedure for innovative products to prevent the aforementioned delays (10, 11). It would be important to ensure that payers do not deliberately delay product entries as cost-containment measures, even if the importance of drug market access from day one is still questionable: significant for new breakthrough medicines but uncertain for me-too drugs.

Implementing the pharmaceutical policies of the United Kingdom in all other countries showed a substantial budget decrease in most of the countries, especially due to policies encouraging generic discount and penetration. Unsurprisingly, it was Germany that experienced the most important decrease. This might be explained by the fact that in the United Kingdom uptake of innovative drugs is delayed compared to that in Germany. The level of access to drug remains less restricted in Germany compared to the United Kingdom, even if the free pricing system would be replaced by the early benefit assessment (all new drugs eligible for early benefit assessment are freely priced at launch for 12 months). In the United Kingdom, the system is more focused on cost-containment and efficiency, with restricted access to the drug until the NICE guidance or Scottish Medicines Consortium (SMC) advices are issued. While this scenario led to an important impact in terms of savings, and highlighted a huge possibility for savings on drug budget of selected EU countries, it might not be the most desirable approach to reduce pharmaceutical expenditure. The low profit margins for generics, the low distribution margins, and the restricted drug access characterizing the UK system might not necessarily fit the culture and healthcare organization of non-UK countries and might not be sustainable for companies manufacturing generic and branded drugs. Finally, applying a reimbursement rate to 100% would have an important economic impact in most of the countries, making this scenario infeasible.

Branded products being the main component of pharmaceutical expenditure, a scenario implementing price cuts on branded products was not tested, due to its obvious outcome.

## Conclusions

This research evidences how forecasting pharmaceutical expenditure is a critical exercise. It can inform policy decision makers and help them foresee the potential risk of ending outside the budget approved by their parliament. It is of primary importance that policy makers can rely on robust models to support their decisions. This work pinpoints a number of opportunities for payers to generate savings on pharmaceutical expenditures that might potentially be used to fund new innovative treatment options. The most important leverages identified by the model were the price, penetration rate, and distribution of generic and biosimilars. Reducing, even slightly, the prices of generics had a significant impact on savings. However, very aggressive pricing of generic and biosimilar products might make this market unattractive and be counterproductive. Implementing policies to promote the use of generics and biosimilars would allow boosting generic/biosimilar penetration, thus increasing savings. Delaying time to access for innovative products was also identified as an effective leverage to increase savings. It is already used by some countries to reduce the pharmaceutical bill, but it might not be a desirable policy for breakthrough products. The important costs incurred by expanding the national pharmaceutical expenditure coverage, suggested that patient financial contributions either directly or indirectly via their private insurances are likely to increase.

## Footnotes

1 Net budget impact is the difference between additional costs of new drug entries and savings associated to generic/biosimilar drug penetration.

2 National government websites (publication of applicable laws), National drug agencies, European Generic Association (EGA) website, Country-specific Pharmaceutical Industries Associations websites, Country-specific Generic Associations websites (as well as direct contacts with some of them).
